# On the Origin of Interoception

**DOI:** 10.3389/fpsyg.2016.00743

**Published:** 2016-05-23

**Authors:** Erik Ceunen, Johan W. S. Vlaeyen, Ilse Van Diest

**Affiliations:** ^1^Research Group on Health Psychology, Faculty of Psychology and Educational Sciences, University of Leuven, LeuvenBelgium; ^2^Research Group on Self Regulation and Health, Institute for Health and Behaviour, Integrative Research Unit on Social and Individual Development, FLSHASE, University of Luxembourg, WalferdangeLuxembourg

**Keywords:** interoception conceptualization, body state perception, phenomenological experience, cross-modal integration, subjective experience

## Abstract

Over the course of a century, the meaning of interoception has changed from the restrictive to the inclusive. In its inclusive sense, it bears relevance to every individual via its link to emotion, decision making, time-perception, health, pain, and various other areas of life. While the label for the perception of the body state changes over time, the need for an overarching concept remains. Many aspects can make any particular interoceptive sensation unique and distinct from any other interoceptive sensation. This can range from the sense of agency, to the physical cause of a sensation, the ontogenetic origin, the efferent innervation, and afferent pathways of the tissue involved amongst others. In its overarching meaning, interoception primarily is a product of the central nervous system, a construct based on an integration of various sources, not *per se* including afferent information. This paper proposes a definition of interoception as based on subjective experience, and pleas for the use of specific vocabulary in addressing the many aspects that contribute to it.

## Introduction

While interoception is a term that has gained and still is gaining popularity in the academic literature since the start of the millennium, consensus on its meaning is as yet not fully established. What is generally agreed upon by most current scholars is that interoception is the perception of the state of the body. The exact interpretation of this definition ranges from the original restrictive meaning which is still adhered to by some (e.g., Dworkin, 2007) to the now more commonly used very inclusive meaning (e.g., Craig, 2002; Wiens, 2005). The restrictive meaning holds that only sensations stemming from viscera are interoceptive. However, throughout this review interoception is used in the inclusive sense; as an umbrella term for the phenomenological experience of the body state, an experience which is ultimately a product of the central nervous system (CNS), regardless of what information the brain uses and does not use to construct this experience. Arguments supporting this choice will be addressed and elaborated upon later throughout different parts of this review.

The relevance of interoception in its inclusive meaning will be illustrated by briefly highlighting its range of involvement across a spectrum of different areas of psychology and health. Next, the importance of proper communication on interoception will be stressed, regardless of the definition one has. The original meaning of interoception will be examined and a short overview of the linguistic development of interoception and related concepts over time will be provided. Finally, an impetus will be given for applying a clear vocabulary that allows to distinguish between the various aspects which can contribute to interoception, while retaining the use of an overarching term. This review will end with some concluding remarks.

### Scope of Relevance

While not yet common parlance in medical circles, “interoception” is a concept which relates to a very wide range of health related and psychological aspects of human life, playing a role in every individual. As a consequence, interoception is of pivotal importance to a wide range of research, theory and translational applications of research findings. A cursory glance at the literature is sufficient to see that interoception relates to a vast range of subjects. These subjects include pain (Craig, 2003), medically unexplained symptoms (MUSs; Bogaerts et al., 2010; Schaefer et al., 2012) as well as medically identifiable symptoms (Julius et al., 2002; Mandelzweig et al., 2006; Janssens, 2011), negative emotions (Pollatos et al., 2007); anxiety, anxiety disorders, and affective disorders (Barlow et al., 2004; Domschke et al., 2010; Dunn et al., 2010b; Paulus and Stein, 2010; Stern, 2014), emotions in general (James, 1884; Lange, 1885; Schachter and Singer, 1962; Damasio, 1994; Wiens, 2005; Craig, 2008; Zaki et al., 2012; Damasio and Carvalho, 2013), emotion regulation (Füstös et al., 2012), decision making (Damasio, 1994; Bechara et al., 1997; Paulus, 2007; Clark et al., 2008; Dunn et al., 2010a, 2012; Paulus, 2011), subjective time perception (Craig, 2009; Pollatos et al., 2014), subjective (self)awareness and consciousness (Craig, 2004; Seth et al., 2011), food and water intake (Berthoud, 2006; Herbert et al., 2012a; Brannigan et al., 2014), eating disorders (Pollatos et al., 2008; Herbert and Pollatos, 2014), addiction (Paulus et al., 2009; Naqvi and Bechara, 2010; Verdejo-Garcia et al., 2012), sexual functioning (Everaerd et al., 2006; Gerbarg and Brown, 2011), empathy (Singer et al., 2009; Fukushima et al., 2011), meditation (Farb et al., 2012), hypnosis (Woody and Szechtman, 2007), and of course interoceptive conditioning (Razran, 1961; Pappens et al., 2013).

Although this list is unlikely to be exhaustive, and though it is beyond the scope of this review to specify for each of these subjects how they relate to interoception, it should be clear that interoception is not to be considered a minor field of study within psychology and health, and that its study has widespread relevance. The focus of this review is to address the semantics of interoception.

#### Interoceptive Conditioning

Classical and operant conditioning are worth further elaboration, as this spearheaded research on interoception. Ivan Pavlov is most known for first describing conditioning on dogs who learned to salivate in response to the sound of a bell [conditioned stimulus (CS)], after they had learned that the bell predicted food [unconditioned stimulus (US)] would come. It is also Pavlov who emphasized that not only exogenous stimuli such as the bell, but also endogenous events, i.e., changes in the ‘milieu interieur,’ could serve as CS (Pavlov, 1927). This idea formed the basis for what is termed interoceptive conditioning: conditioning where either the CS, the US, or both are subjectively perceived as informative of the body state (Razran, 1961). Interoceptive conditioning has been hypothesized to be of importance in the etiology, maintenance and treatment of chronic pain (De Peuter et al., 2011), functional disorders, cancer related fatigue (Meagher, 2010), hypertension (Koroboki et al., 2010), eating disorders (Davidson, 1993; Oldershaw et al., 2011), mood and anxiety disorders (Paulus and Stein, 2010; Deacon et al., 2013), and drug addiction (Wise et al., 2008; Bevins and Murray, 2011; Bevins and Besheer, 2014; Troisi, 2014).

Studies on the role of interoceptive conditioning in drug addiction provide a useful paradigm for evaluating interoceptive conditioning. Moreover, they provide a paradigm for assessing subjective perception of changes in body state, i.e., interoception. For example, Lubinski and Thompson (1987) describe how two pigeons were conditioned to effectively communicate to each other and three other pigeons whether they were experiencing the effects of a stimulant (cocaine), a depressant (pentobarbital), or no drug (saline). Learning to recognize and communicate these different drug induced body states, generalized to the ability to effectively communicate states induced by another stimulant (d-amphetamine) and depressant (chlordiazepoxide) without need for further training. Likewise, rats can be trained to discriminate drug effects of nicotine from a non-drug state. This learned discrimination can serve to alter their behavior (lever pressing), help to establish the median effective dose necessary for receptor signals to become perceptible, and provides a model for drug use and drug craving. The aforementioned drug discrimination paradigms have demonstrated that it is possible to qualitatively and quantitatively manipulate the subjective body state, and that this altered subjective state due to drugs can serve as CS (Bevins and Besheer, 2014), or rather as an operant discriminative stimulus (Troisi, 2014). Thus, drug discrimination and state dependent learning are forms of interoceptive conditioning. The importance of interoceptive conditioning for a variety of widespread conditions mentioned in the previous paragraph, as well as a useful paradigm for researching interoception, further underscores there is a demand for a clear definition of interoception and related terminology that allows for subtle, but critical distinctions within this larger function based concept.

#### Pain and Interoception

Also among the list of subjects related to interoception (see Scope of Relevance), the subject of pain deserves special mention. The International Association for the Study of Pain (IASP) defines pain as “an unpleasant sensory and emotional experience associated with actual or potential tissue damage, or described in terms of such damage” (Merskey, 1986). While those with a narrow definition of interoception would only consider visceral pain interoceptive, those with more inclusive definitions of interoception such as the one used throughout this review – consider all forms of pain as a form of interoception. This inclusive approach is supported by functional neuroimaging studies which find that the neural network activated during pain and during other forms of interoception are very much the same (Legrain et al., 2011; Moseley et al., 2012). It is also indirectly supported by the well-validated finding that emotion plays a significant role in pain; a role which it also plays in all other forms of interoception. For example, negative emotions impact the affective component of pain negatively and decrease pain tolerance (Carter et al., 2002), while positive emotion increases pain tolerance (Zweyer et al., 2004). In dyspnea one can observe something remarkably similar. In a negative affective context, dyspnea elicits more respiratory symptom report than in a positive affective context (von Leupoldt and Dahme, 2013). This parallel between pain and dyspnea can be taken as an additional indication that pain really is nothing more than a specific form of interoception, but interoception nevertheless. Regardless of which definition of interoception one applies, the inclusion of pain in the definition of interoception -even if merely including visceral pain- has its merit. It provides a fertile soil for cross-fertilization of ideas from the vast area of pain research on the one hand, and that of research on non-painful types of interoception on the other.

### Communicating on Interoception

It is surprising that communication around the concept interoception often stumbles over definitional differences between authors. While both an overarching concept of body perception as well as more specific concepts deserve their own place, the exclusive focus on one approach could lead to undervalue the importance of the other. For example, an overarching concept has as one advantage that it crosses the bridges between different types of research findings. How this overarching concept is labeled, changes over time as do so many aspects of language. In the 19th century this overarching concept was referred to as “coenaesthesis,” in the early 20th century “coenesthesia,” in the second half of the 20th century “somesthesis,” and now in the 21st century it is most frequently referred to as “interoception” (see Semantic Evolution). Regardless of its label, an exclusive focus on an umbrella term may lead to premature overgeneralization of findings.

For example, the accuracy with which to detect heartbeats has long been referred to as a general measure of overall interoceptive accuracy. It is true that interoception includes the ability to perceive heartbeats. However, prior to having tested how accuracy with which to detect heartbeats correlates with accuracy of perception of the heterogeneous plethora of other types of interoceptive sensations, it would be premature to say that all forms of interoceptive accuracy are poor in those who have poor accuracy in perceiving their own heartbeat – even if this hunch later appears to be correct. Such a conclusion would remain premature until the point where there are actual findings which support the conclusion that heartbeat accuracy can be generalized to reflect accuracy of perception of other interoceptive sensations. This caution has also been emphasized by the authors previously (Ceunen et al., 2013a). After submission of the letter to the editor cautioning against premature generalization, Herbert et al. (2012b) published a research article which does suggest that accurate cardioceptive perceivers also have more accurate perception of sensations that aid in the regulation of water intake. Findings like these allow to gradually make evidence-based extrapolations, and stimulate speculations on how generalizable findings are. At the same time, we need to remain careful not to overgeneralize beyond what has been investigated, nor to blindly accept extrapolations suggested by others as facts.

Arguments referring to neuroanatomy are sometimes used to justify generalizations such as cautioned against in the previous paragraph. One example of such a generalization is referring to the convergence of sensory information from distinct bodily organs and of distinct types of visceroceptors in the nucleus of the solitary tract (NTS). Though processed in very close proximity to each other within the NTS and sometimes in overlapping areas, the respective loci in the NTS necessary for each of these sensations are still somewhat distinct (Paton et al., 1999). Also, convergence at one site of processing does not necessarily mean that at all other levels of processing there is not a single distinction between any of these distinct body state sensations. Therefore, to prevent oneself from being tempted into premature generalization, it is useful to keep in mind that different sensations not only overlap in some aspects (e.g., all being interoceptive and activating a largely common neural network), but are also unique in their own ways. This uniqueness of sensations holds true even if all these sensations have in common that they refer to the body state and have at least one or more common processing sites in the brain. Examples of levels at which sensations can be considered as being distinct from one another can be: whether sensations are entoperipheral or epiperipheral in origin; whether they are exogenous or endogenous; whether the individual does or does not experience a sense of agency over what causes these sensations; whether the sensations stem from tissues with efferent autonomic nervous system (ANS) and enteric nervous system (ENS) or from somatic nervous system (SNS) innervation; whether the concerned organs are identical or different in their ontogenetic origin as coming from ectoderm, endoderm, or mesoderm; and which afferent homeostatic pathways and basal processing structures are involved in the early processing. I will go into more detail on a number of these in separate sections later on (see Exogenous versus Endogenous Origins; Visceroceptor, Visceroceptive, Visceroception – A Reference to Efferent Innervation; The Homeostatic Afferent Pathways and Early CNS Processing of Homeostasis). The main point is that, even though there may be commonalities between different body state sensations, there are also always differences. Interoceptive sensations are characterized by both specificity and convergence. Though an overarching term can be beneficial, this all goes to emphasize that we should always keep in mind to avoid premature generalization that has not first been specifically addressed in research.

On the other end of the spectrum, as opposed to the exclusive focus on an overarching concept and an exclusive focus on similarities, there can be an exclusive restrictive focus on one of the subcomponents of interoception; on what makes this subcomponent different from other sensations. Just as the broad focus on similarities, so too does the narrow focus on differences have its own advantages and disadvantages. An advantage of research with a narrow focus is that this will provide specific information on a subcomponent of interoception, which can then be contrasted to other subcomponents of interoception, which allows us to make statements on the generalizability or specificity of these and other findings. A disadvantage of a more narrow focus is that at times, findings from certain research areas do not provide, nor receive input from distinct yet related research areas. As a result the information from different types of studies is less likely to be put together to form a more complete picture.

To allow for better communication of not only findings, but also for cross-fertilization in the sharing and forming of ideas and insights on interoception, and on its relation to human psychological faculties and health related issues, there needs to be a common understanding amongst researchers. To achieve this, it is imperative that the various components of the concept interoception are outlined so they become more generally acknowledged as distinct, individual aspects which each deserve their own specific labels, while at the same time there remains a concept which integrates all of these aspects. That labels are prone to change over time, that they differ between certain research areas, and that there are individual differences in the use of these labels only makes an outline of this topic more relevant. Although this review is not written on the pretense of being able to create a universally accepted consensus on which labels to use, it does intend to at least provide an impetus for the use of distinct labels for the distinct aspects of interoception that will be covered here. Moreover, this review emphasizes that it is every author’s duty to introduce their own however short definition of interoception for each individual publication, and to make sure it matches with how they use the word throughout that publication.

## History of a Concept

### Etymology

To come to a deeper understanding of the meaning of a word, or concept, it is customary to refer to its origins and then address whether, and if so, how its meaning has changed over time. Interoception is a relatively recent concept which arose together with the concepts proprioception and exteroception during the early 20th century. The first known usage of the concept interoception in publication dates to Sherrington (1906) in his book “The Integrative Action of the Nervous System,” which is a collection of lectures he had given at an unknown date prior to publication. In the book, Sherrington talks of “interoceptors,” “interoceptive receptor fields,” “interoceptive reflex arcs,” “interoceptive surface,” and “interoceptive segments.” Interestingly, at this point in time, the noun “interoception” itself was not yet introduced in publication. In fact, it is only in the 1940s that the word “interoception” first appears in scientific journals (Freeman and Sharp, 1941; Mogendovich, 1941; Airapetyantz and Bykov, 1945). Regardless, we do need to refer back to Sherrington to understand the original meaning of the concept interoception.

Sherrington referred to the internal surface of the body as interoceptive, as opposed to exteroceptive which he defined as the external surface in direct contact with the environment. In this meaning interoceptive then can be considered a synonym for things entoperipheral, while exteroceptive is a synonym for things epiperipheral. Thus, according to this definition, cutaneous sensations would be considered exteroceptive sensations, but subcutaneous not. In the vast entoperiphery, Sherrington further distinguished between deep somatic tissue, i.e., skeletal muscle, as a site specific to proprioceptors, and the viscera as site specific to interoceptors. Furthermore, he considered not only perception of light, sound, odor, and mechanical touch as exteroceptive, but also perception of temperature and nociception. The inclusion of temperature and nociception in the definition of exteroception, contrasts to these sensations being included in more recent definitions of interoception, such as the one put forward by Craig (2002). In Sherrington’s (1906) definition, what distinguished interoception (and proprioception) from exteroception is that only the latter possesses the quality of projicience. Projicience is a term which he used to refer to two aspects: (1) the perception of something at a distance outside of our body (exogenous), and (2) projection in the sense of estimating the future (precurrent) based on what is happening now. In other words, Sherrington labeled perception of precurrent exogenous stimuli as exteroceptive, while sensations of endogenous origin as either proprioceptive or interoceptive, depending on whether they arise in respectively skeletal muscles or viscera.

While the linguistic contraction of “interior receptor” was the basis for “interoceptor” and by extension the adjective “interoceptive,” in contrast the noun “interoception” was first introduced more than a third of a century later, and can either be taken to be a variation of the contraction of “interior receptor,” or to be a new contraction, namely “interior perception.” Whatever, interoception’s original meaning, modern day use of “interoception,” and to some extent “interoceptive” have generally come to refer to the broader phenomenological perception, rather than to refer merely to location and stimulation of receptors. In other words the focus of the concept has shifted from referring solely to the afferent relay of receptors of the ANS, to becoming a word which is now most frequently used as an umbrella concept for a multi-sensory, multimodal integrated percept of the body state.

### Semantic Evolution

In order to identify the frequency and evolution of usage of specific interoception related labels, an extensive search in Google Scholar was performed. The inclusion of selected terms for which a frequency of occurrence was obtained, was motivated by the idea that at some point in time all of these included terms have had a nearly synonymous meaning to one of our primary three search entries: interoceptor, interoceptive, interoception. In addition to these three words, the search entries included the following: visceroceptor, visceroceptive, visceroception, somesthesis, somesthetic, somesthesia, coenesthesis, coenesthetic, and coenesthesia. The frequency of each of these words was established by identifying the number of search results from 1800 up to and including 2010, with the “include patents” and “include citations” boxes unchecked. The number of hits per word was assessed per period of 5 years (1901 up to and including 1905, 1906–1910, 1911–1915, etc.), excepted the period from 1800 up to and including 1900, a period which was taken as a whole.

Moreover, the same search procedure was conducted for variations of each of these words to allow for identifying possible changes in spelling preference over time and to identify the first introduction of alternate spellings. The authors identified and conducted a separate search for alternate spellings, which were: interoreceptor; interoreceptive, interoperceptive; interoreception, interoperception; visceroreceptor; viscero receptive, visceroperceptive; visceroreception, visceroperception; somaesthesis, somataesthesis, somatesthesis; somaesthetic, somataesthetic, somatesthetic, somathestetic; someaesthesia, somataesthesia, somatesthesia; caenesthesis, caenaesthesis, coenaesthesis, coenoesthesis, cenesthesis, cenoesthesis; caen esthetic, caenaesthetic, coenaesthetic, coenoesthetic, cenesthetic, cenoesthetic; caenesthesia, caenaesthesia, coenaesthesia, coeno esthesia, cenesthesia, cenoesthesia.

Because Google Scholar identifies and includes some alternate spellings or concepts automatically in the search results it produces, this could potentially create the problem of getting wrong estimates. This problem was bypassed by entering each individual search entry between brackets so only hits for the specified spelling resulted. Another aspect taken into consideration is that in Google Scholar, when the number of results is higher than 10, the total number of hits as indicated at the first page of search results is usually merely an initial approximation by the Google Scholar search engine, but does not always correspond exactly to the total number of hits. The correct number of hits is indicated on the last page of results (if the total is less than approximately 950 hits). Therefore, in order to get a more accurate approximation of the exact amount of hits, the total number of hits as indicated on the last accessible page of results was used. For those hits ranging over 1000 with 10 hits per page, Google Scholar does not display pages beyond approximately the 95th page, so approximations of the total number of hits when there are more than approximately 950 results in total may be less accurate.

Although it is beyond the scope of this review to present the collected data in extensive detail, a selection has been made of aspects which stand out and provide an interesting perspective on the development of word preferences (see **Table 1**). During the entire span of the 19th century, interoception was not yet an existent word. Instead, with a total of 220 results for its various spellings in that period, ‘coenesthesis’ was by far the most popular word which comes closest to the inclusive meaning of interoception, followed in popularity by ‘coenesthesia,’ which in its various spellings totals only 11 results for that same period. While ‘somesthesis’ and ‘somesthesia’ appear to be non-existent in publication during the 19th century, the adjective ‘somesthetic’ did exist in publication starting around the late 1800s (totaling five results up until 1900). Bailey (1906) was responsible for introducing the word ‘somesthesis’ into the English language, in the same year that Sherrington (1906) published the first work to use the words ‘interoceptor’ and ‘interoceptive.’ Two years later, it was again Bailey (1908) who first introduced the word ‘somesthesia.’ About a century later, in the period from 2006 up to and including 2010, ‘somesthesis’ and ‘coenesthesia’ are still relatively popular nouns, with respectively 284 and 263 publications in which these words appear, but both are very much overshadowed by the popularity of the noun ‘interoception’ with mention in 1745 sources. It is true that in that 5 years period from 2006 up to and including 2010, the adjective ‘somesthetic’ also occurs in a large number of sources (1579 sources to be precise), but this seems to be largely due to the use of the adjective as a synonym for ‘somatosensory’ when referring to the CNS areas SI and SII. Regardless of the reason for its popular use, the occurrence of the adjective ‘somesthetic’ in recent years is still by far outdone by the adjective ‘interoceptive,’ the latter which occurred almost five times as much as the former, in a total of 7471 sources.

**Table 1:** Word preferences over the centuries.

	Year of first mention (author)	Most popular time relative to ‘equivalent’ words	Publications from 2006–2010
Interoceptor	Sherrington, 1906	1906–2010	202
Visceroceptor	Strong and Elwyn, 1948	/	18

Interoceptive	Sherrington, 1906	1981–2010	7471
Visceroceptive	MacLean et al., 1952	/	170
Somesthetic	Taylor and Haughton, 1900	1897–1900;	1579
*^∗^Somaesthetic^∗^*	*^∗^Barker, 1897^∗^*	1906–1910;	
		1931–1980	
Coenesthetic	Kellogg, 1901	1858;	194
*^∗^Caenaesthetic^∗^*	*^∗^Noble, 1858^∗^*	1901–1915	

Interoception	Freeman and Sharp, 1941; Mogendovich, 1941	2001–2010	1745
Visceroception	Dutov, 1974	/	103
*^∗^Visceroreception^∗^*	*^∗^Merkulova and Popova, 1967^∗^*		
Somesthesis	Bailey, 1906	1936–1940; 1946–2000	284
Somesthesia	Bailey, 1908	/	205
Coenesthesis	De Hájnik, 1816	1794–1910	53
*^∗^Caenesthesis^∗^*	*^∗^Hübner, 1794^∗^*		
Coenesthesia	de Nyir, 1817	1911–1935; 1941–1945	263

*Time during which words were most popular is derived by the number of mention relative to ‘equivalent’ words. Such ‘equivalent’ words are grouped together between two horizontal lines. For those words where the now most widely used spelling arose only later, alternative spellings are included and marked with an asterisk*.

While it is obvious how this data set can provide insights on the development of word usage, it may be unclear how it can shed light on the development of word meaning over the years, hence a clarification for the latter is in order. It has already been pointed out that in their initial existence, the words ‘interoceptive’ and ‘interoception’ were more narrowly defined concepts (Sherrington, 1906; see Etymology). Craig (2002) made a plea to consider interoception as a more overarching term. Since then that particular publication has been cited in well over 2000 other publications, and the usage of the word interoception has spiked in popularity. If we then reconsider the collected word prevalence data, it appears as if interoception is most frequently used from the point in time onward when a broader meaning was first attributed to it. The increase in popularity after this point onward is in part due to at least two important factors. First, an initial suggestion toward a conceptual shift likely leads to a lack of consensus and thus increased mention and use of the word in attempts to reach consensus, or in attempts to think through, clarify and solidify its meaning. Secondly and most importantly, concepts with broad meanings have broader relevance to a variety of research lines, whereas concepts with narrow meanings have relevance to a more limited number of research areas. Following this logic, we can assume that words increase in popularity at least in part because their meanings shift to refer to a broader concept. (However, we cannot conclude the reverse: that when words decline in popularity, it is because their meanings have narrowed down. It is possible for words to decline in popularity simply because other words are attributed a similar meaning and have become more popular.)

The arguments outlined here justify two choices made in this review. First and most important, is that a choice has been made to adopt and extend on the use of the word interoception in its broad, overarching meaning, rather than try to revert back to its originally restrictive meaning. I.e., this review builds on the already existing conceptual change that has occurred after the original inception of the words ‘interoceptive’ and ‘interoception’ last century. Secondly, given that currently interoception is the most widely used word from all previously indexed, related concepts, it justifies the choice for the title and focus of this review to be on the words interoception and interoceptive to refer to the broader perception of the body state, and to use related definitions to more specifically classify sensations.

## Aspects of Interoception

This review argues in favor of using the word interoception as an overarching concept. We make a plea that anything which falls within this larger concept, or which is related but different, ought to be labeled differently and more specifically. Doing so helps to avoid confusion and allows for more effective communication. Whether there is consensus on the labels is only of secondary importance, as meaning attributed to labels will naturally evolve over time. Of primary importance is to establish a consensus that each of the concepts listed below deserve their own labels and are not to be confused with one another, even though they may be related to one another.

### Exogenous versus Endogenous Origins

If something has an exogenous origin, this means that the source originates or is attributable to an agent outside the organism. If something is endogenous, it means it comes from within the organism and is not attributable to an external agent. It is clear that ‘exogenous’ and ‘endogenous’ are antonyms of one another. Likewise, exteroception is commonly accepted to be the antonym of interoception. Therefore, how exteroception is defined, to some extent affects the meaning attributed to interoception. This can be somewhat problematic, as there has been a conceptual shift in the meaning of interoception, while the meaning of exteroception has hardly changed for most who use it. The resulting problem this poses for the definition of interoception is twofold.

The first problem relates to the meaning attributed to exteroception, namely that it is the sensory perception of exogenous stimuli. This meaning is often interpreted to mean that all sensations elicited by exogenous stimuli are exteroceptive, and considers the actual stimulus origin of primary importance and not the subjective perception arising in the CNS. This approach implies that any experimental set-up that intends to study interoception, would only be able to do so if sensations would have an endogenous origin. This would largely preclude the study of interoception and would have to discard many of the published studies on the topic, as nearly every stimulus applied in lab set-ups has an exogenous origin. In other words, confounding exogenous and endogenous origins with the phenomenological experience of something as relating to the surrounding environment or to the body state, would seriously set back the study of interoception, and conclusions made on the topic of interoception. Furthermore, many naturally occurring body state sensations are very frequently elicited by exogenous stimuli. For example, gastro-intestinal sensations can follow the ingestion of exogenous substances. Likewise, the sensation of feeling cold is not necessarily of endogenous origin as in illness, but can just as well have an actual exogenous cause such as a cold ambient temperature. As the human body does not act in isolation of its surroundings, it is necessary to keep concepts that make a distinction between the origins of a stimulus (exogenous versus endogenous) distinct from broader concepts that make a distinction between different types of experiential perception as arising in the CNS (interoception versus exteroception).

The second problem posed by referring to exteroception as an antonym of interoception is that this often leads to the assumption that the receptor systems and pathways for both must by definition be mutually exclusive. However, that is not necessarily the case when using the inclusive definition of interoception. For example, seeing and feeling snow in the absence of cold sensations, can lead to the perception that the perceived snow, although not imaginary, is not genuine snow. This exteroceptive percept is the result from an integration of various sensory modalities including body state sensations (as well as past experience and other factors). If we imagine a nearly identical scenario but accompanied by sensations typical of physical illness, this can give a whole new phenomenological feel to the absence of cold sensations, where this absence can then be integrated in the interoceptive perception of the body state rather than the exteroceptive perception of the surrounding environment. This example, though hypothetical, illustrates that exteroception and interoception can rely on identical sensory receptors and afferent pathways, and that they need not be mutually exclusive on any of the levels preceding the higher order processing of interoception and exteroception.

The main point of this section is that sensory origin or stimulus properties (exogenous versus endogenous) are not of relevance to determining whether a percept is interoceptive or exteroceptive when using the inclusive definition of interoception. What matters in the inclusive definition is whether a sensation is experienced as informative about the body state or about the surroundings (see Interoception as Integrated Percept). In those cases where the actual origin of a sensation is considered of relevance for research purposes or conclusions, rather than the phenomenological experience, it is preferable to refer to the eliciting stimuli as exogenous or endogenous (whichever is applicable), and give preference to the use of these terms over ambiguous terminology.

### Visceroceptor, Visceroceptive, Visceroception – A Reference to Efferent Innervation

Although not in popular usage yet (see **Table 1**), in this review it is argued that there is a place for the words visceroceptor, visceroceptive, and visceroception. These labels have become more suitable to refer to the once restrictive concepts that “interoceptor,” “interoceptive,” and “interoception” originally referred to, i.e., things specifically and solely pertaining to the viscera and nothing else (Sherrington, 1906). Such a distinction is necessary as interoception has come to adopt a more broad meaning, which refers to the integrated cross-modal CNS perception of the body state (Craig, 2008; Critchley and Harrison, 2013). Distinguishing between the broad concepts “interoceptive,” and “interoception” on the one hand, and the narrow concepts “visceroceptive” and “visceroception” on the other, helps to avoid all possible confusion between the broad and the specific. Moreover, given the broad meaning of interoception, it can be argued that any receptor that can provide information to create a CNS representation of the body state can be considered an interoceptor, and not just those receptors in the viscera. This makes the word interoceptor so inclusive it becomes redundant (“receptor” would be sufficient). At the same time this necessitates the use of a more specific label for referring to only those receptors located in visceral tissue. Hence, it is proposed here to refer to these visceral receptors as “visceroceptors” rather than “interoceptors.” In the same vein the adjective “interoceptive” and the noun “interoception” should be solely reserved for more broad meanings pertaining to perception of the body state when further details are not necessary or when the focus is on generalities. In contrast, the adjective “visceroceptive” and the noun “visceroception” are encouraged to be used when referring specifically to visceral tissue origins, distinct from and not including somatic tissue origins.

Of course, it is crucial then that there is understanding of what viscera are, because, as is the case with the word interoception, there is more than one definition. We can recognize at least three types of definition: (1) one arbitrarily grouping certain anatomical structures under the label viscera, (2) another based on efferent innervation, and (3) a final one focusing on perceptual differences.

#### The Dictionary Definition

One definition used for distinguishing viscera from somatic tissue creates this divide as based on anatomical location, and is commonly found in dictionaries; it either labels (a) only the intestines, or either (b) all intra-thoracic, intra-abdominal, and intra-pelvic organs as viscera (Berube et al., 2008). The problem with considering only the intestines, i.e., the part below the stomach as viscera, is that the stomach and organs located in the thorax can then neither be considered visceral, nor somatic – yet no other label is provided for these “gray zone” body tissues. As for the anatomy based definition which considers all intra-abdominal and intra-thoracic organs to be viscera, it simply classifies the remainder of the body as somatic tissue. That is: not only skin and skeletal muscles, but also joints and bones (Lewis, 1938). This is usually accompanied by a further arbitrary subdivision of somatic tissue distinguishing the skin from the remaining “deep” somatic tissue. Whether, the circulatory system is visceral or somatic according to any such anatomical definition usually remains unmentioned, as the circulatory system branches out into all areas of the body, making it difficult to classify based on its location. Also, if viscera are strictly those organs located in the trunk, then the female reproductive system should be considered to be entirely visceral, whereas at least part of the male equivalent (in addition to the dermis) should be considered somatic. As no such claims are made by anyone, this implies that the anatomy based definition as given is not strictly adhered to even by its proponents, and that the dictionary definition is not sufficient by itself to classify tissues. For better communication, it is considered preferable to use definitions which do not leave any room for subjective interpretation and which do not require additional, implicit, unmentioned criteria.

#### Definition as Based on Efferent Innervation

In contrast to the aforementioned dictionary definition, there exists a very straightforward, clear-cut physiology based definition that makes the distinction between visceral and somatic tissue as based on actual efferent innervation (Wolfsohn, 1914). Relying on existing knowledge of efferent SNS innervation and ANS innervation to determine which tissues are respectively somatic and which ones visceral, deserves preference for two reasons.^1^ First, it does not leave a single tissue of the entire body unmentioned, and would classify the circulatory system as visceral (Livingston, 1935). Second, it does not leave room for arbitrary individual choices on which organic tissues to include under the label viscera, and which ones not, because physiologically verifiable, existent efferent innervation cannot be contested.

Note should be taken that making the distinction as based on efferent innervation differs on some important aspects from those who simply label all organs in the trunk as viscera and consider the remainder of the body as somatic. First, when basing ourselves on efferent innervation, we can determine that the skeletal system is in fact to be labeled as visceral, and not somatic (Kini and Nandeesh, 2012). One implication of this is that bone pain thus is to be considered a visceral, and not a somatic pain according to innervation. Another implication is that, in so far that sensory feedback from the skeletal system (including periosteum) contributes at all to proprioception, this would then be a visceral component contributing to the CNS representation of the body in space (proprioception), which is perfectly possible if we adhere to inclusive definitions of proprioception, interoception and exteroception, where all that matters is the phenomenological experience and not which type of receptors are involved in creating that experience.

Other differences between the dictionary definition and the efferent based definition for distinguishing somatic from visceral tissue relates to the classification of the skin, the esophagus, and the respiratory system. If we consult known information on efferent innervation, we can conclude the skin is in fact not a purely somatic tissue in contrast to what is often stated (Oaklander and Siegel, 2005; Gibbins, 2013). The skin actually contains both SNS as well as ANS innervation, making it a partially somatic, partially visceral organ. In psychophysiology this visceral aspect is well-recognized, where dermal autonomous changes such as changes in pilo-erection and sweat secretion can be and are used to assess physiological aspects of emotion (Dawson et al., 2007; Benedek and Kaernbach, 2011).

Like the skin, the esophagus is a single functional unit, yet its proximal section has SNS innervation, whereas the distal section has ANS innervation, also making the esophagus an organ which is partially somatic, and partially visceral. Unlike the skin, the esophagus has a clear division between the visceral and somatic parts. Also included in this list of mixed SNS and ANS innervation, although not strictly speaking an organ, is the respiratory system (Kuntz, 1944).

All three of the aforementioned – the skin, the esophagus, and the respiratory system – have a prominent role in the interoception literature and related research. Craig was the first to argue that some tactile sensations, such as sensual touch, are distinct from other touch sensations and are relayed to the brain together with other homeostatic sensations (Craig, 2002). While Craig based his argument on afferent innervation (see The Homeostatic Afferent Pathways and Early CNS Processing of Homeostasis), this review makes the distinction between visceral and somatic based on efferent innervation, and uses another label for afferent based differences. The esophagus too is gaining increased attention in interoception research, as it allows to distinguish between visceroception (if stimulated distally, i.e., the lower part) and somatoception (if stimulated proximally, i.e., the upper part; Aziz et al., 2000; Ceunen et al., 2015). As for the respiratory system, since early human experience it has been the gateway to altering and gaining control over ANS function and thus control over the viscera (Sovik, 1999). In fact, one of the first written records where breathing is considered to be able to affect the viscera, is in a book by Tao Hongjing, written sometime around the end of the 5th or start of the 6th century, where six different methods of breathing are considered beneficial to the health and functioning of six different viscera (Zhang et al., 2007). Because respiration can be used to increase the inotropic output of heart rate (Shannahoff-Khalsa and Kennedy, 1993), which in turn increases heartbeat perception accuracy (Herbert et al., 2012a), respiration may even be considered to be a gateway for altering heartbeat perception and perhaps also for altering other forms of visceroception.

#### Definition as Based on “Typical” Sensory Properties

A final note on methods for distinguishing between somatic and visceral tissues concerns sensory properties. The viscera are often attributed three typical sensory properties that are thought to distinguish them from somatic tissue. These three visceral properties are said to be (1) the inability to volitionally bring visceral sensations into awareness, (2) poor discrimination of sensations, and (3) poor localization (Ray and Neill, 1947; Aziz et al., 2000; Dunckley et al., 2005; Dworkin, 2007). Each of these points will be addressed here.

As to the first point, although visceral sensations generally only enter awareness bottom–up (e.g., when homeostasis is disrupted, or in mental disorders), it is actually possible to volitionally attend in a top–down method to visceral sensations as is done in meditative practices. Even though such increased awareness does not necessarily imply increased accuracy (Khalsa et al., 2008; Ceunen et al., 2013a), nevertheless it is possible to volitionally attend to visceral sensations, which underscores that the first property associated with visceral sensations is not correct.

The second sensory property often associated with viscera, namely poor discrimination, i.e., poor perceptual accuracy, is indeed very common to most ANS innervated organs, yet not universally applicable to all. For example, there are subgroups of individuals who can very accurately perceive their heartbeat. One may argue such accurate heartbeat perception is possible due to heartbeats resonating in somatic muscle tissue overlying the heart region, thus implying there is not sufficient evidence for the existence of accurate visceroception. However, even when sensations from overlying somatic tissues are absent, accurate heartbeat detection is still possible (Khalsa et al., 2009), which indicates that good discrimination is possible even for at least one type of visceroception, and perhaps also for other types.

As for poor localization, it is true that the majority of visceral sensations are phenomenologically experienced as vague, diffuse, or pertaining to a general area, rather than to a precise spot. However, from an experience level, pain stemming from kidneys, appendix, genitalia, and anus all have known instances where these were subjectively experienced as sharp, and/or in a clearly localized way (Boyle, 1997; Bajwa et al., 2001; Kafkia et al., 2011; Sejdinovic et al., 2011; Sandella et al., 2012; Fauconnier et al., 2013), although none of these pains stem from tissues innervated by SNS efferents. Moreover, tactile sensations including itch, sensual touch and temperature can be fairly accurately localized. Taking into consideration that the skin is partly ANS innervated, this is yet another example which questions whether all visceral sensations are indeed poorly localized. Furthermore, note should be taken that not all somatic tissue sensations are characterized by accurate localization either (Lewis, 1938; Feinstein et al., 1954).

#### Implications

One practical implication is that, when classifying body tissues as either somatic or visceral, it is suggested here not to combine classification as based on sensory properties with classification as based on efferents: this is simply not completely accurate and is confusing to the critical reader. An example of one such to be avoided, confusing statement would be: “we consider this organ as visceral BECAUSE it is ANS innervated and BECAUSE its sensations are poorly localized.” Instead, it would be better to say: “We consider this organ as visceral because it is ANS innervated. Sensations from most, but not all ANS innervated organs, including/excepted this one, are poorly localized.” When sensory properties are considered truly relevant to specific research conclusions or predictions, distinguishing viscera with sensory properties typical to most viscera, from other viscera with anomalous sensory properties may definitely have its value. E.g., one could make the distinction between those typical ANS innervated organs that are always poorly discriminated and/or poorly localized, as opposed to those few ANS innervated organs which can at times be accurately discriminated and/or accurately localized. (Accurate discrimination refers to heartbeat detection through visceral afferents only; accurate localization, albeit not always, refers to kidneys, appendix, genitals, anus, and the tactile sensations – the latter being included because the skin is partly ANS innervated.)

In practical, research oriented terms, this section clarifies how to classify stimuli as either visceral or somatic in such a way that if others apply the same method of classification, they will make exactly the same conclusions as to which body tissues are visceral and which are somatic. Moreover, this section also implies that stimulation of certain organs or systems can elicit sensations which are a combination of both visceral and somatic components. For example, respiratory stimuli such as loaded breathing (i.e., breathing against a resistance) and CO_2_ inhalation have as visceral component the sensory feedback from the lungs and the CO_2_ levels in the circulatory system, while the somatic component is the sensory feedback from the respiratory muscles (Epstein et al., 1995). Likewise, cold pain as induced by the cold pressor test (a test where subjects are required to submerge their hand in cold water), is not a purely somatic stimulus as is often suggested by those who interchange the terms “somatic,” “exteroceptive,” and “exogenous” as synonyms. Immerging the hand in cold water in fact affects visceral tissue in addition to somatic tissue. It does so through the baroreflex which involves the (visceral) circulatory system, but also because the skin is an organ with both visceral and somatic components, rather than being purely somatic. Moreover the cold penetrates beyond the skin, not only into the somatic muscle tissue beneath the skin, but potentially penetrating as deep as into the bones, for which there even is an English expression, namely “being cold/chilled to the bone.” Even without being literally cold to the bone, cold pain as induced by the cold pressor test has been known to have been subjectively perceived as radiating from the submerged hand all along the veins across the entire lower arm, as reported by participants in an earlier study of the authors (Ceunen et al., 2013b). Such subjective experience of ‘spreading’ cold suggests that pain induced by the cold pressor is at least partly dependent on visceral sensory feedback stemming from the circulatory system, as described by Livingston (1935).

The most important implication of this section for research purposes, is that under natural circumstances visceral sensations are frequently accompanied by somatic sensations as is the case for heartbeats, and possibly also for ingestion of large amounts of food. Therefore, it is imperative that before designing an experiment, the researcher determines whether it is absolutely crucial to elicit visceral sensations without eliciting any somatic sensations whatsoever, or whether ecological validity is more important. If the aim is merely to elicit a sensation that resembles a real life sensation as close as possible, it may not be necessary to come up with elaborate, time consuming or other effortful investments intended to annihilate any possible somatic sensation from co-occurring with a visceral sensation. Moreover, these contraptions intended to block out somatic sensations can create other (albeit constant) variables into an experimental set-up, affecting the outcomes of a study just as much, but merely in a different way than when such ‘precautions’ would not have been taken.

#### Overview of “Visceroceptor, Visceroceptive, Visceroception – A Reference to Efferent Innervation”

In summary, this section argues in favor of labeling visceral and somatic sensations specifically when needed, rather than invariably lumping them under the more generalized terms interoception and exteroception, and it also argues that interoception and exteroception are not synonyms for respectively visceral and somatic. Although, it is true that sensations arising from viscera are most often contributing to the phenomenological interoceptive percept, in certain instances visceral sensations can potentially contribute to the phenomenological exteroceptive percept of what is going on in the environment around us. It was further argued that viscera are preferentially to be defined as those organs with efferent ANS innervation, while somatic tissues are to be defined as body tissues with efferent SNS innervation. It has been brought to the reader’s attention that some organs such as skin and esophagus, or functional units such as the respiratory system have a combination of both types of innervation. Furthermore, although poor discrimination and poor localization are common to most ANS innervated organs, it is inaccurate to conclude that an organ must be somatic simply because sensations thereof can be accurately perceived and/or well-localized. In other words, it is incorrect to state that poor localization and poor discrimination are a universally defining characteristic that allow to determine whether an organ is to be considered ANS or SNS innervated. Using efferent innervation to guide our definition allows for physiologically based conformity across researchers in determining which kind of sensations are to be considered visceral, which ones somatic, and which ones a combination of both. Also of note is that in designing experiments where ecological validity is the aim, attempts at creating ‘purely’ visceral sensations may not even be necessary, although these may be informative.

### The Homeostatic Afferent Pathways and Early CNS Processing of Homeostasis

When Bud Craig redefined interoception as the sense of physiological status of all tissues of the body, he specified this “sense” as being a CNS representation, while at the same time arguing that this representation starts at the receptor site and is relayed via what he labeled as the homeostatic pathway (Craig, 2002). While Craig labels both the relay of the homeostatic state of the body (from receptor site up to primary levels of processing) and higher order levels of processing as interoceptive, we prefer to use two different labels to distinguish these two aspects. Specifically, we would reserve interoception to refer to the higher order processing, which occurs once the mid-insula gets involved (see Interoception as Integrated Percept). The process from receptor site up to primary areas of processing are labeled here as homeostatic pathways (see The Homeostatic Afferent Pathways and Early CNS Processing of Homeostasis). Take note that there is not just one homeostatic pathway, but instead there are at least three to five (Critchley and Harrison, 2013) depending on how one counts, as will be outlined in the paragraphs immediately hereafter.

**FIGURE 1:**
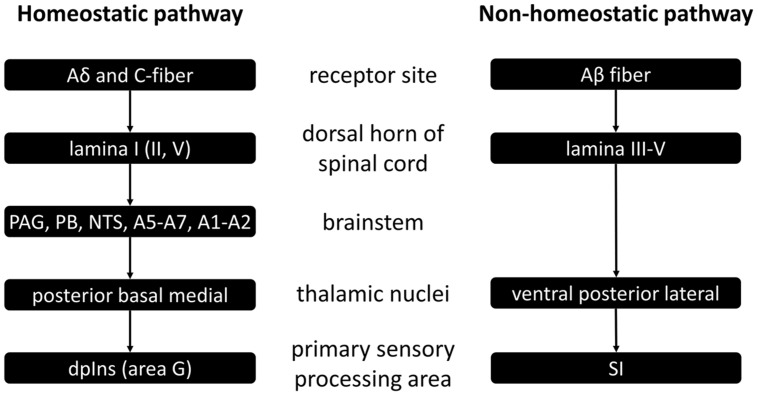
**Schematic representation of spinal homeostatic and non-homeostatic afferent pathways as based on (Craig, 2010)**. PAG, periaqueductal gray; PB, parabrachial nucleus; NTS, nucleus tractus solitarii a.k.a. nucleus of the solitary tract; dpIns, dorsal posterior insula; SI, primary somatosensory cortex.

The spinal homeostatic pathway refers to all processes as illustrated in **Figure 1**, prior to mid insula involvement (Craig, 2010). It starts with stimulation of Aδδ and/or C-fiber receptors, is relayed via the first (and also the second and fifth) lamina of the dorsal horn of the spinal cord, on to the brainstem homeostatic regions which form a pre-cortical homeostatic representation, and then to the posterior, basal and medial thalamic nuclei. Finally there is activation of the primary sensory processing area for homeostatic sensory input, namely the dorsal posterior insula. This spinal homeostatic pathway is distinct from the spinal relay of non-homeostatic sensory information, which is schematically depicted on the right hand side of **Figure 1**, and of which discussion in further detail is beyond the purpose of this review.

Other than (1) the spinal homeostatic pathway, there are also other routes via which primary homeostatic processing can occur (Critchley and Harrison, 2013). There is the (2) cranial homeostatic pathway. It is that of the cranial nerves, such as vagus and glossopharyngeal nerves carrying information from the receptor sites to the brainstem – first to the NTS, and then on to the parabrachial nucleus (PB) and periaqueductal gray matter (PAG) – and from there on to thalamus, hypothalamus, amygdala, and ultimately to the anterior cingulate cortex (ACC) and the insula. It may be of interest to note here that taste – often categorized as one of the distinctly non-interoceptive sensations – is in fact relayed via cranial nerve afferents (Craig, 2005).

Then there is also (3) the humoral homeostatic pathway, which reaches the CNS via circulating substances. The humoral pathway refers in fact to at least three different pathways of information transduction, which all share the commonality that they are in first instance activated via circulating substances. The (a) ventricular (or classical) humoral pathway detects changes in substances present in the third and fourth ventricles, and first engages the circumventricular organs which are located adjacent to these ventricles; these include the area postrema (AP), the organum vasculosum of lamina terminalea (OVLT), and the subfornical organ (SFO). The humoral info processed here in turn projects to the NTS, the hypothalamus, the PB, sympathetic medullary nuclei, the dorsal motor nucleus, the nucleus ambiguous, midline thalamic nuclei, and again the insula and ACC. The (b) blood–brain (or non-classical) humoral pathway is that which detects changes in those substances passing the blood–brain barrier. It involves the NTS, hypothalamic nuclei, the medial amygdala nucleus, and monoamine systems, and can influence the information relay between ventral striatum, insula, and cingulate. The (c) microglial (or extraneuronal) humoral pathway is that in which the microglia in the circumventricular organs, leptomeninges and choroid plexus respond to peripheral presence of pathogens and inflammation. The changes taking place in these microglia in response to these signs of infection and tissue damage activate a cascade of microglial activation across the CNS.

### Interoception as Integrated Percept

**FIGURE 2:**
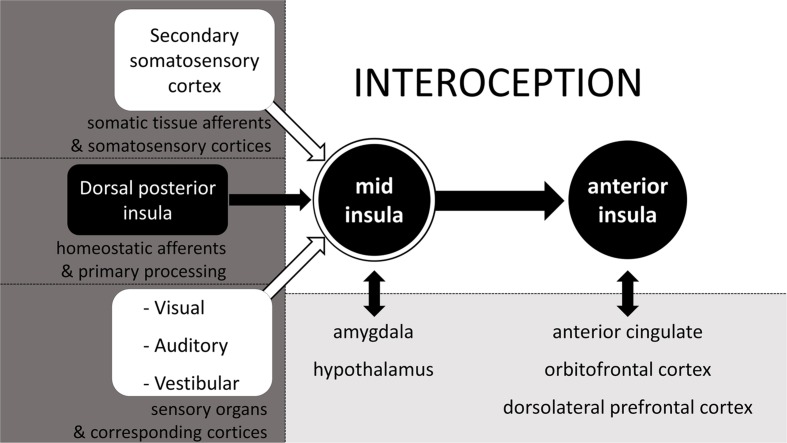
**The neurophysiology of interoception**. Schematic simplification of the neurophysiology behind the cross-modal integrated (re)representation of the body status, otherwise known as interoception. Adapted from Craig (2008).

Although, the spinal, cranial, and humoral homeostatic pathways are the most direct routes of sensory feedback concerning the status of all tissues and the state of the body, they are not exclusive contributors to the highest order percept of the body status. In constructing a central, higher order representation of the body status (i.e., interoception) the CNS relies on all available information, which it integrates in the mid insula (Craig, 2008). Other than homeostatic feedback relayed from the dorsal posterior insula (or primary interoceptive cortex) to the mid insula, the mid insula also receives input from the secondary somatosensory cortex, thus effectively allowing for the integration of spinal non-homeostatic afferent information (see **Figure 1**) in the interoceptive percept. In addition, also visual, auditory and vestibular feedback are integrated at the mid insula (Craig, 2008). The mid insula further communicates with and integrates information from the amygdala regarding stimulus salience and emotional memories, as well as with the hypothalamus regarding current state of the ANS and of ongoing metabolic processes (see Figure 2, adapted from Craig, 2008). Thus, the mid insula is considered to be the locus responsible for the integrated re-representation, feature extraction and cross-modality integration, i.e., the core structure needed for what can be considered interoception (Craig, 2010). When this integrated re-representation is relayed to the right anterior insula where also subjective time perception is processed, interoception enters the realm of apperception, i.e., conscious interoception. As can be seen in **Figure 2** from the presence of reciprocal connections represented by two-way arrows, as well as is indirectly evident from section 2.2.3, arriving at this higher order integrated percept involving the mid insula and the anterior insula, is in fact not an entirely sequential hierarchical process (Critchley and Harrison, 2013). Although there is a posterior-to-mid-to-anterior processing in the insula, there is also a lot of cross-talk between many of the “lower” areas with one another as well as cross-talk from these areas from and to the mid insula and the anterior insula. As such, interoception is in fact the sum total of all structures involved in addition to activation of mid and anterior insula – it is the product of a neural matrix for body state perception (Craig, 2005; Legrain et al., 2011; Moseley et al., 2012; Critchley and Harrison, 2013).

Examples from the subjective perception of body state will be given here and in paragraphs immediately following this one, to illustrate the part that each of the sensory inputs as illustrated in **Figure 2**, contribute to interoception. In **Figure 2**, we can see that somatic sensations relayed via the secondary somatosensory cortex can potentially contribute to interoception. Although, the ability to accurately perceive heartbeats does not necessarily require afferent feedback from somatic tissue (Khalsa et al., 2009), in cases were such afferents are involved, there is activation of somatosensory areas, which results from sensations relayed via the spinal non-homeostatic pathway (see **Figure 1**). It is likely that such clear somatic sensations are most common with inotropic activation of the heart, as manipulations that increase inotropic output, also increase cardioceptive accuracy (Herbert et al., 2012a).

As seen in **Figure 2**, vision can play a significant role in interoception. This contrasts to popular views, wherein vision is generally considered to be a sensory faculty which solely contributes to the perception of the surrounding environment. An example where a visual experience is part of interoception is the gray-out that often occurs shortly before the onset of syncope. For the individual, subjectively this gray-out is part of the cascade of sensations of the fainting experience and of the percept that the homeostatic status of the body is disrupted (Kamiya et al., 2005; Shukla and Zimetbaum, 2006). Another well-accepted finding that supports and illustrates the notion that vision can in fact contribute to the perception of the body status comes from satiety research, in which visual feedback has long been recognized as one of the factors affecting food intake, a basic homeostatic function crucial for survival (Berthoud, 2006; Morton et al., 2006; Cornier et al., 2007). Moreover, visual feedback also affects the experience of acute pain and phantom limb pain (Ramachandran and Rogers-Ramachandran, 1996; Chan et al., 2007; Mancini et al., 2011), which further underscores that vision has the potential to contribute to the perception of the own body state. Even the sight of facial expressions of others can affect processing of information about the own body state – at least for pain (Mailhot et al., 2012; Khatibi et al., 2014; Wieser et al., 2014).

Not only vision has the potential to contribute to interoception. Auditory information or the disruption or absence thereof can just as much contribute to the phenomenological interoceptive percept. The most obvious example is tinnitus, which can indicate either the onset of syncope, inner ear damage, or which can be part of a set of symptoms which indicate some sort of homeostatic disruption (McGuinness and Harris, 1961; Bitterman, 2004; König et al., 2006; Shukla and Zimetbaum, 2006; Nam et al., 2010). Tinnitus also includes instances of actually hearing one’s own heartbeat, which is referred to as pulsatile tinnitus and which can go together with high blood pressure or other circulatory abnormalities (Mattox and Hudgins, 2008). Of course not all forms of pulsatile tinnitus correspond to the heart-rate, e.g., when pulsing is caused by spasms of ear muscles, but even then the pulsatile tinnitus reflects a physiological abnormality, i.e., deviation from the homeostatic state of the body. Moreover, verbal information can also alter perception of the body state and affect it, for example under social stress or during hypnosis (Darwin, 1872; Drummond et al., 2003; Tan et al., 2005). All these are mere examples which indicate auditory feedback can and does at times contribute to the perception of the body state.

As for the potential of vestibular sensory information adding to interoception, many may have experienced it as the feeling of dizziness or vertigo which sometimes accompanies physical illness and therefore can be indicative of it. From all these examples, it should be clear that really any type of sensory information, and not merely that from homeostatic pathways can get integrated into the overall body percept. It is only because of this integration of sensory input that biofeedback is possible in the first place and can help in the treatment of many psychiatric disorders in which interoception plays a major role (Schoenberg and David, 2014). Of course the selection of examples listed here are by no means exhaustive of how non-homeostatic sensory information can contribute to interoception: they merely serve as illustrations of how multisensory interoception truly is.

Note also that interoception is defined as a cross-modal integrated representation of the body status, rather than merely a multisensory representation. It is cross-modal because this phenomenological experience of the body status not only integrates input from a variety of peripheral sensory channels, but also integrates information from, and cross-talks with different structures within the CNS as can be seen in **Figure 2**. Much of the information relayed via the sense organs which gets integrated in the interoceptive percept, can only be integrated because learning provides the opportunity to identify these percepts as informative on the body state. This learned integration can be effected via conditioning or other forms of learning (e.g., Pappens et al., 2013; Bevins and Besheer, 2014). Seth (2013) proposes that interoception, or interoceptive inference as he labels it, is not just passive, bottom–up processing, but is something which also involves active top–down activation to make predictions of the causes of sensory input. His view is based on the central idea of predictive coding, which is that perception is a process of not only afferent feedback, but also of predictions, and ultimately the integration of both, resulting in prediction errors. Predictive coding models also consider MUS as arising from not only peripheral sensory feedback, but also from prior beliefs, where attention, attributed agency, expectation, prior experience and even cultural beliefs all play a role in perception of symptoms (Edwards et al., 2012). This is clear for example from the effect of instruction in decreasing (placebo) or increasing (nocebo) visceral pain intensity (Schmid et al., 2013). More support for the argument made here, is that interoception can be manipulated by something as simple as categorizing interoceptive sensations versus rating those same sensations on a continuous dimension (Petersen et al., 2014). The change in interoception with this sort of experimental manipulation is likely effected via a mechanism of biasing perceptual decision making, as shown extensively in categorization research involving exteroception. Further supporting the argument of CNS involvement in body state perception are corollary discharge models of effort, which hold that the perception of physical exertion is entirely centrally generated, rather than resultant from somatic afferents (Marcora, 2009). Taken together, we should at least consider the possibility that body state perception other than perception of effort may also be in part centrally generated.

It has been suggested by Paulus and Stein (2010) that with increased ambiguous or noisy sensory input from the homeostatic pathways and decreased accuracy of perception, the brain relies especially on itself (as well as on alternative sensory channels) enhancing top–down modulation and creating a self-referential biased percept of what is going on with the body. Whether, individuals with somatoform disorders, mood disorders and anxiety disorders are less or more visceroceptively accurate is hard to conclude given opposing research findings. That people with these disorders are not entirely homogenous with regard to their ability for heartbeat detection, can be related to findings from McGrath et al. (2013). Their findings suggest that at least for major depression, and perhaps for mood and other disorders, there are two subgroups of patients: one group consists of individuals with an overactive anterior insula, and the other of individuals with an underactive anterior insula. These two distinct neurological biomarker patterns of these two subgroups are suggestive respectively of accurate and inaccurate perceivers. This would explain why findings regarding cardioceptive accuracy in the aforementioned disorders are contradictory. Regardless of the accuracy with which individuals with mood and anxiety disorders can perceive sensory homeostatic afferent feedback, individuals with such disorders excessively rely on sources other than actual bottom–up homeostatic pathways, giving more weight to maladaptive cognitive-emotional schemes of interpretation (Paulus and Stein, 2010). All of the aforementioned further contributes to the view that the perception of the body state, i.e., interoception, is a truly multimodal percept.

## Conclusion

Although, Sherrington (1906) originally came up with and used the label interoceptive as a synonym for things visceral, over the course of time, interoception has come to mean much more than just that. While interoception is sometimes referred to as viscerosensory integration (Critchley and Harrison, 2013), interoception is more than the central sensory integration of afferents stemming from only the viscera. Interoception has in fact come to refer to a multimodal integration not restricted to any sensory channel, not restricted to mere sensations, but also relying on learned associations, memories, and emotions and integrating these in the total experience which is the subjective representation of the body state. Interoception defined as such includes any form of pain, not just visceral pain, but somatic pain as well.

This inclusive definition of interoception is not new. Rather, this review expands on this view and the formerly made association with the inclusion of pain in this definition. It is guided by the most commonly accepted definition of pain to serve as inspiration for the definition of interoception. Pain is defined as based on its phenomenological experience rather than referring to the physical origin of the pain sensation or any physiologically objectively quantifiable aspect (Merskey and Bogduk, 1994). The IASP considers pain to be a psychological state, and although it recognizes that pain often has a proximate physical cause such as a noxious agent activating nociceptors and nociceptive pathways, it emphasizes that this need not always be the case, and that therefore the presence or absence of a noxious stimulus is not relevantin determining whether there is pain or not, and neither is the activation of nociceptors or nociceptive pathways.

Like this definition of pain then, so too has “interoception” become such a broad concept that it has been argued here that interoception should be defined as a subjective experience of the body state. Although in many instances, this experience may well be elicited by a peripheral change in homeostasis, this need not necessarily always be so. Independent of the phenomenological experience which is interoception, aspects potentially contributing to interoception can be classified in myriad ways. One way is to consider whether sensations have an endogenous or exogenous origin. Whatever, the actual source of a sensation, endogenous or exogenous, it does not determine whether a perceived sensation is to be considered interoceptive or not. The only thing determining whether something is interoceptive is whether it contributes to the subjective perception of body state. The same can be said of the distinction between somatic tissue and viscera. Although it is often relevant to distinguish visceral from somatic tissue, it does not mean sensations stemming from somatic tissue cannot contribute to the phenomenological percept of the status of the body. To avoid confusion between visceral sensations on the one hand, and the subjective feeling state that is interoception on the other, it is suggested in this review to not use these two related but distinct concepts as synonyms. In particular, it is preferable to keep words which contain a direct linguistic reference to viscera (e.g., viscerosensory, visceroceptive, visceroceptor, visceroception) reserved for instances where the distinction between ANS/ENS efferent innervated tissue on the one hand and SNS innervated tissue on the other is of equal or more relevance than the sum phenomenological experience of the general state of the body.

Homeostatic pathways (including early CNS homeostatic processing) have also been discussed in this review, and are considered to provide the most direct sensory feedback on the state of the body. The authors prefers to label these as homeostatic rather than interoceptive pathways, and only speak of interoception from the point in processing onward where there is a higher order integration of information, sensory and neural, taking place to form a body state representation in the CNS. Thus, the “-ception” in “interoception” is taken to no longer refer to “reception” (i.e., receiving) of stimulation, but rather the CNS “perception” of the body state. Perception itself is always an inherently flawed and subjective reconstruction of reality by the CNS, never an accurate one-to-one representation. Hence, the core of the definition of interoception is on the subjective experience above all else; thus we can say the brain is the true source, i.e., the real origin of interoception.

In summary, in this review we have critically examined the *origin* of interoception in two major ways, and conclude with a statement on that which we argues is the true origin of interoception. In first instance, (1) the *etymological origin* of the word interoception has been investigated, and in extension thereof its semantic development too. This approach, apart from clarifying why there is a lack of consensus, has provided the ground from which we were able to logically distill the various components related to and contributing to interoception. Each of these various components has at one point been considered to be (2) the *physical origin* of interoception: some considered the stimulus the origin of interoception (endogenous versus exogenous), other the organs involved (viscera versus somatic tissue), still others the homeostatic pathways through which the signals are transmitted. Although these may all contribute to interoception and affect our experience, none of these are essential to interoception, because it is in fact the CNS, where perception is created, and so it is inside our heads that we may find *the very origin of our interoceptive experience*.

## Author Contributions

All authors listed, have made substantial, direct and intellectual contribution to the work, and approved it for publication.

## Conflict of Interest Statement

The authors declare that the research was conducted in the absence of any commercial or financial relationships that could be construed as a potential conflict of interest.

## Footnotes

1 Although, the authors of this review recognize that the enteric nervous system (ENS) has its own reflex activity independent from the ANS, both ANS and ENS innervated tissue are classified here under the label “visceral” as opposed to “somatic.”
